# An Electrochemiluminescence Biosensor for the Detection of Alzheimer’s Tau Protein Based on Gold Nanostar Decorated Carbon Nitride Nanosheets

**DOI:** 10.3390/molecules27020431

**Published:** 2022-01-10

**Authors:** Roghayeh Jalili, Salimeh Chenaghlou, Alireza Khataee, Balal Khalilzadeh, Mohammad-Reza Rashidi

**Affiliations:** 1 Research Laboratory of Advanced Water and Wastewater Treatment Processes, Department of Applied Chemistry, Faculty of Chemistry, University of Tabriz, Tabriz 51666-16471, Iran or r.jalili@tabrizu.ac.ir (R.J.); salimehchenaghlou@gmail.com (S.C.); 2 Department of Environmental Engineering, Gebze Technical University, Gebze 41400, Turkey; 3 Department of Material Science and Physical Chemistry of Materials, South Ural State University, 454080 Chelyabinsk, Russia; 4 Stem Cell Research Center (SCRC), Tabriz University of Medical Sciences, Tabriz 51666-14711, Iran; khalilzadehb@tbzmed.ac.ir

**Keywords:** gold nanostars, Tau protein, immunosensor, carbon nitride nanosheets, Alzheimer’s disease

## Abstract

Human Tau protein is the most reliable biomarker for the prediction of Alzheimer’s disease (AD). However, the assay to detect low concentrations of tau protein in serum is a great challenge for the early diagnosis of AD. This paper reports an electrochemiluminescence (ECL) immunosensor for Tau protein in serum samples. Gold nanostars (AuNSs) decorated on carbon nitride nanosheets (AuNS@g-CN nanostructure) show highly strong and stable ECL activity compared to pristine CN nanosheets due to the electrocatalytic and surface plasmon effects of AuNSs. As a result of the strong electromagnetic field at branches, AuNSs showed a better ECL enhancement effect than their spherical counterpart. For the fabrication of a specific immunosensor, immobilized AuNSs were functionalized with a monoclonal antibody specific for Tau protein. In the presence of Tau protein, the ECL intensity of the immunosensor decreased considerably. Under the optimal conditions, this ECL based immunosensor exhibits a dynamic linear range from 0.1 to 100 ng mL^−1^ with a low limit of detection of 0.034 ng mL^−1^. The LOD is less than the Tau level in human serum; thus, this study provides a useful method for the determination of Tau. The fabricated ECL immunosensor was successfully applied to the detection of Tau, the biomarker in serum samples. Therefore, the present approach is very promising for application in diagnosing AD within the early stages of the disease.

## 1. Introduction

Alzheimer’s disease (AD) is the most prevalent neurodegenerative illness. The number of people suffering from the disease will increase—with the rapidly aging world population. It is expected that AD is going to affect 115 million people worldwide if no effective therapeutic strategies are developed by 2050. AD-related pathophysiological symptoms can be observed as early as 10–20 years before the emergence of cognitive impairment [1,2,3]. Most of the AD biomarkers appear in the cerebrospinal fluid (CSF). However, the CSF sampling route is difficult, expensive, and invasive, making it inconvenient for large-scale or repetitive sampling in a long-time following disease progression or treatment efficacy. Human Tau protein is a promising serum biomarker for AD—that has gained significant attention for early and accurate molecular diagnostics [4,5,6,7]. However, the serum concentrations of Tau protein biomarkers are at the pg/mL level, and ultrahigh-sensitivity detection methods are in great demand for clinical applications.

Electrochemiluminescence (ECL), also called electrogenerated chemiluminescence, converts the input electrical signal into optical readout, which is different from most other electrochemical methods [8,9]. In fact, ECL relies on the generation of luminophore in the vicinity of the electrode surface by applying voltage. Then, these electronically excited luminophores participate in high-energy electron-transfer reactions to emit luminescent signals [10,11]. ECL represents a valuable marriage of electrochemistry and spectroscopy and displays unique advantages over other optical methods, including fluorescence and chemiluminescence (CL). First and foremost, the absence of an excitation pathway not only facilitates the detection setup but also diminishes the fluctuation in the traditional fluorimetric techniques, thus leading to high sensitivity. Second, the advantages of chemiluminescence are maintained in ECL methods. Furthermore, the electrochemical reaction in ECL permits the time and location of the light-emitting reaction to be controlled. This advantage leads to improved sensitivity, specificity, and reliability compared to CL. Third, ECL is more selective than CL because the production of excited species can be easily controlled by varying the electrode potentials [11,12].

Colloidal gold nanoparticles (AuNPs) are extensively utilized as the sensitivity enhancement components of biosensors due to their unique photochemical properties [13]. Recently, anisotropic AuNPs such as nanocubes, nanowires, and nanostars have received significant attention since they have unique and often better optical and electronic properties than spherical NPs [14,15,16]. Gold nanostars (AuNSs) have a circular structure with sharp protruding branches [17,18,19,20]. AuNSs display modulated plasmon resonance because their multiple sharp tips could act as “hot spots” and amplify the local electromagnetic fields considerably. Applications of AuNSs for amplifying response signals of surface-enhanced Raman spectroscopy (SERS) based immunosensors are reported frequently in the literature [21].

At present, the available detection methods for Tau protein are based in large part on the enzyme-linked immunosorbent assay (ELISA). Despite the restrictions of the conventional ELISA, alternative methods have rarely been reported for the detection of Tau in plasma. In a pioneering study, a multichannel surface plasmon resonance (SPR) platform was applied for the detection of human Tau in undiluted plasma [2]. Dan Tao et al. reported a label-free electrochemical aptasensor for the detection of Tau in human serum with a limit of detection of 0.70 picomolar [22]. An electrochemical biosensor was developed on an interdigitated wave-shaped electrode via an activated and self-assembled monolayer to immobilize a specific antibody for the detection of tau at picomolar levels [23]. To our best knowledge, the ECL application has not been reported in Tau protein detection. Furthermore, a combination of localized surface plasmon resonance (LSPR) and ECL can be an effective way to amplify the signal intensity, and LSPR induced PL enhancement has been used as a sensitive analytical technique.

As a semiconductor two-dimensional (2D) material, Graphite-like carbon nitride nanosheets (g-CN nanosheets) have received great attention in both fundamental and applied scientific investigations. g-CN nanosheets possess intriguing advantages, including good biocompatibility, excellent surface area, stability, and medium bandgap, thus extending huge potential applications as ECL emitter candidates. However, surface modification of the electrodes with pure g-CN nanosheets reduces the conductivity of modified electrodes and produces weak and unstable ECL emissions. For improving the ECL properties of g-CN nanosheets, it has been appropriately functionalized with some nanomaterials. For example, an ECL biosensor was constructed based on Au NPs decorated g-CN nanosheets to quantify carcinoembryonic antigen with excellent sensitivity (LOD: 6.8 pg mL^−1^), good specificity, precision, and stability [24]. Our group reported an ECL immunosensor based on AuNSs and g-CN nanosheets for the quantification of CD133 peptide with a LOD of 0.257 ng mL^−1^ [12].

**Scheme 1:**
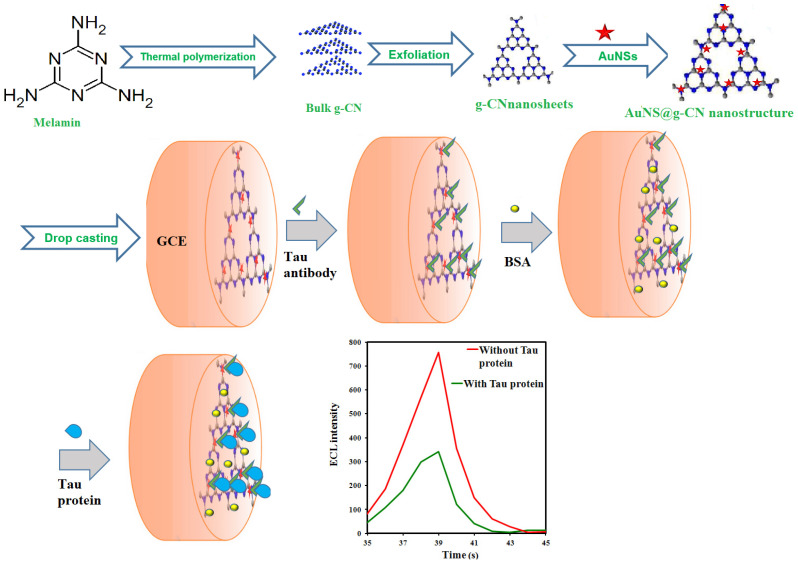
The illustration of the fabrication route and sensing mechanism of the Tau biomarker immunosensor.

In this report, an ECL immunosensor based on an AuNS functionalized g-CN nanostructure (AuNS@g-CN) was developed for Tau protein detection. The construction procedure of the suggested ECL immunosensor for Tau protein is displayed in Scheme 1. The AuNS@g-CN nanostructure generated a stable and strong ECL emission. AuNSs not only accelerated electron transfer in the ECL process but also served as the immobilization platforms for biomolecules. Hence, the AuNS@g-CN nanostructure was combined with the Tau antibody via the Au–N and Au–S bonds and produced a selective immunosensor by specific recognition between the antibody and the antigen (Tau protein). The ECL response is linear in the 0.1 ng mL^−1^ to 100 ng mL^−1^ of Tau protein concentration ranges, whit LOD of 0.034 ng mL^−1^. The real sample test demonstrated that the ECL method had good practicability for sensitive Tau protein determination in serum samples. Thus, this method could prove valuable in diagnosing AD within the early stages of the disease.

## 2. Experimental

All details of materials, procedures, and instruments are described in the Electronic Supplementary Materials (ESM).

### 2.1. Synthesis of g-CN Nanzosheets

Bulk CN was prepared according to the previous procedure through the pyrolysis of melamine in the air atmosphere [25]. In detail, a crucible containing 15 g of melamine was placed in a Muffle Furnace. The important parameters of calcination were adjusted as follows; Initially, the temperature was increased to 500 °C with the heating rate of 2.5 °C min^−1^ and kept at this temperature for 5 h; Finally, the temperature cooled down to room temperature naturally to obtain the yellow bulk g-CN powder. Nanosheets of g-CN were obtained by a liquid exfoliating technique. First, 50 mg of bulk g-CN product was suspended in 50 mL of deionized water and sonicated for 30 min (150 W) by an ultrasonic cell disruptor. In the next step, the g-CN suspension was exposed to ultrasound in an ultrasonic bath (400 W) for 10 h. The resultant white suspension was centrifuged at 5000 rpm for 30 min to remove the residual un-exfoliated g-CN or large-sized nanosheets.

### 2.2. Synthesis of Gold Nanostars

Gold nanostars (AuNSs) and AuNPs were synthesized as described in the literature [26]. Details are given in the ESM.

### 2.3. Preparation of the AuNS Decorated g-CN Nanosheets (AuNS@g-CN Nanosheets)

AuNS@g-CN nanostructures were produced by the physical mixing of 1 mL of AuNSs suspension into 20 mL of g-CN nanosheet suspension (dropwise) and stirred gently overnight. Finally, the resultant suspension was centrifuged, and the obtained product was washed with water successively. The resulting AuNS@g-CN nanostructures were centrifuged and dispersed in PBS (pH = 7.4).

### 2.4. Fabrication of ECL Immunosensors

A glassy carbon electrode (GCE) was polished repeatedly with alumina powder on a polishing pad and then rinsed thoroughly with water and ethanol. Finally, polished and clean GCE was dried using nitrogen in a gas stream. Next, 10 µL of the suspension of AuNS@ g-CN nanostructures was directly drop-casted on the cleaned GCE and kept at 40 °C for 30 min to form a thin film of AuNS@ g-CN nanosheets. In the next step, the resultant AuNS@ g-CN nanosheet immobilized GCE was incubated with 10 μL of a Tau antibody (anti-Tau) (7.14 ng mL^−1^) solution and incubated at 4 °C for 12 h. The covalent immobilization of the anti-Tau takes place via the covalent bond formed between the thiol and amine groups of the antibody and AuNSs. Afterward, (GCE/AuNS@ g-CN nanosheets/anti-Tau) electrode was immersed in 0.1 M PBS to remove physically absorbed anti-Tau. To block possible remaining active sites, the electrode was immersed in 10 μL of 1% BSA solution at 37 °C for 1 h and then washed by 0.1 M PBS thoroughly. The immunosensor was applied for the quantitative analysis of Tau protein. The ECL assays were performed as follows: First, 10 μL of Tau protein with certain concentrations was cast on the modified electrode and left to react with immobilized anti-Tau. During the detection process, the specific sites of anti-Tau are identifed and connected to Tau protein when Tau peptide is present in the detection system (GCE/AuNS@ g-CN nanosheets/anti-Tau/BSA/Tau protein), resulting in the ECL signal. The ECL measurements were carried out in 0.1 M PBS (pH = 7.5) and 0.1 M of K_2_S_2_O_8_.

## 3. Results and Discussion

### 3.1. Synthesis of AuNS@g-CN Nanostructures

**Figure 1:**
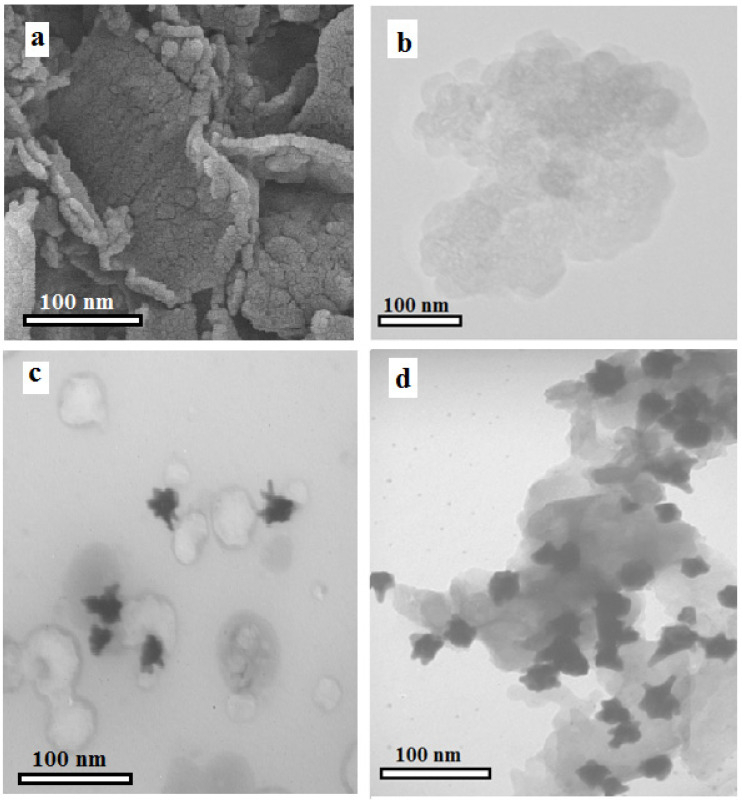
(**a**) SEM image of g-CN nanosheets, (**b**) TEM image of g-CN nanosheets, (**c**) TEM image of AuNSs, and (**d**) TEM image of AuNS@g-CN nanostructure.

In this work, g-CN nanosheets were used as ECL emitters for the fabrication of an immunosensor. The pyrolysis procedure was utilized to construct bulk g-CN. Then, the liquid phase exfoliation technique was applied to obtain nanosheets with one or a few layers [27]. The SEM (Figure 1a) and TEM (Figure 1b) images of g-CN nanosheets indicate that the g-CN nanosheets have a lamellar structure and an average diameter of around 200 nm.

AuNSs were prepared using a surfactant-assisted seed-mediated approach. The morphological characterization of AuNSs was carried out using TEM images (Figure 1c), showing that as-synthesized AuNSs have monodisperse distribution with an average diameter of approximately 20 nm, and distinct spikes with a length of about 5 nm. The AuNSs@g-CN nanostructure was synthesized via a physical mixing route. As shown in the TEM image of the AuNS@g-CN nanostructure (Figure 1d), the AuNSs were consistently distributed on the g-CN nanosheet surface, with no aggregation of the AuNSs. The g-CN nanosheets are also clearly observed in TEM images.

To confirm the impact of the shape of the AuNSs on ECL amplification, the spherical AuNPs-modified g-CN nanosheets (AuNP@g-CN nanostructure) were prepared by physically mixing AuNPs with an average size of (30 nm) and g-CN nanosheets. As displayed in the TEM image of the AuNP@g-CN nanostructure (Figure S1), AuNPs were dispersed evenly throughout the g-CN nanosheet.

The UV-Vis spectra of suspensions of AuNSs, g-CN nanosheets and AuNS@g-CN nanostructures were used to prove the proper synthesis of the nanostructure. The AuNSs exhibit two plasmon resonance peaks (Figure S2a), located at around 550 nm and 700 nm. The locations of the UV-Vis peaks are consistent with AuNSs with the same size as in previous works [26]. The suspension of g-CN nanosheets shows a distinct absorption peak at 320 nm (Figure S2b, red curve). The composite of AuNSs@g-CN nanosheets (Figure S2b, blue curve) displays both typical UV-Vis bands of g-CN nanosheets and AuNSs, indicating that AuNSs are successfully decorated in the g-CN nanosheet structure, and the plasmonic properties of AuNSs are well maintained.

### 3.2. ECL Behaviors and Feasibility

**Figure 2:**
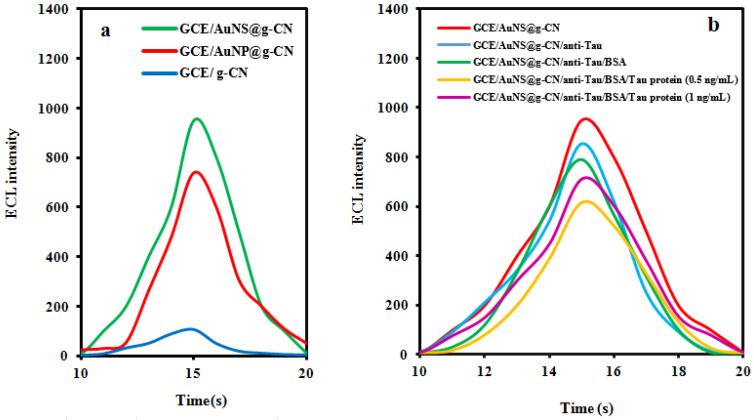
(**a**) ECL intensity–time curves of GCE/pristine g-CN nanosheet (blue curve) system, the GCE/AuNP@g-CN nanostructure system (red curve) and GCE/AuNS@g-CN nanostructure system (green curve), and (**b**) ECL intensity–time curves of GCE/AuNS@g-CN system (red curve), GCE/AuNS@g-CN/anti-Tau system (blue curve), GCE/AuNS@g-CN/ anti-Tau/BSA system (green curve), GCE/AuNS@g-CN/anti-Tau/BSA/Tau protein system (Tau protein: 0.5 ng mL^−1^, purple curve) and AuNS@g-CN/BSA/Tau protein system (Tau protein: 1 ng mL^−1^, yellow curve). (concentration of K_2_S_2_O_8_: 0.1 M, pH = 7.5 and concentration of PBS: 0.1 M).

The ECL performance of the pristine g-CN nanosheet, AuNS@g-CN, and the AuNP@g-CN nanostructure was evaluated by immobilizing the nanomaterials onto the polished GCE and scanning the potential from −0.2 to −2 V. Simultaneously, the ECL intensities were recorded in PBS (pH = 7.5) and a 0.1 mM K_2_S_2_O_8_ solution. As displayed in Figure 2a, the g-CN nanosheet/GCE system (blue curve) shows weak ECL intensity.

When the potential was swept to more positive values, g-CN nanosheets were reduced to g-CN^−^ Nanosheets due to charge injection. The reduction of S_2_O_8_^2−^ generates the strong oxidant SO_4_^−^ which is reduced by g-CN^−^ Finally, the produced excited g-CN* emits luminescence in the electrochemical cell. The relevant ECL reactions are given below:

$$g\text{-}\mathrm{CN}+e^{-}\to g\text{-}{\mathrm{CN}}^{-}$$

$$S_{2}O_{8}^{2-}+e^{-}\to {\mathrm{SO}}_{4}^{2-}+{\mathrm{SO}}_{4}^{-}$$

$$g\text{-}{\mathrm{CN}}^{-}+{\mathrm{SO}}_{4}^{-}\to g\text{-}{\mathrm{CN}}^{*}+{\mathrm{SO}}_{4}^{2-}$$

And/or

$$g\text{-}\mathrm{CN}+{\mathrm{SO}}_{4}^{-}\to g\text{-}{\mathrm{CN}}^{+}+{\mathrm{SO}}_{4}^{2-}$$

$$g\text{-}{\mathrm{CN}}^{+}+g\text{-}{\mathrm{CN}}^{-}\to g\text{-}{\mathrm{CN}}^{*}+g\text{-}\mathrm{CN}$$

$${g\text{-}\mathrm{CN}}_{}^{*}\to g\text{-}\mathrm{CN}+h\nu$$

The ECL intensities obtained using AuNP@g-CN and AuNS@g-CN nanostructure systems (Figure 2a, red and green curves, respectively) were enhanced by 7 and 9 times, respectively, higher than that of the g-CN nanosheet/GCE system (Figure 2a, blue curve). The mechanism of enhancement is attributed to the catalytic effect of AuNPs on the S_2_O_8_^2^^−^ reduction process [24]. Moreover, the more effective enhancement of AuNSs systems from the special shape is due to the concentrate electron density around the spikes. In addition, AuNSs show a strong LSPR effect concentrated around the tips of branches which remarkably amplified the ECL intensity by producing more charge carriers. This enhancement mechanism has been reported in the literature [28,29].

The ECL intensity of different steps was investigated to consider the stepwise assembly procedure of the constructed immunosensor (Figure 2b). The bare GCE modified with AuNS@g-CN nanosheets (GCE/AuNS@g-CN) showed a distinct cathodic ECL emission observed at a potential around −1.2 V (blue curve), indicating good electron conductivity of the GCE/AuNS@g-CN electrode [30,31]. Afterward, with the immobilization of anti-Tau (GCE/AuNS@g-CN/anti-Tau) and BSA (GCE/AuNS@g-CN/anti-Tau/BSA), the ECL intensities displayed a significant decrease due to an obvious decrease in conductivity (respectively blue and green curves), suggesting the successful immobilization of these non-conductive biomolecules on the electrodes. When the GCE/AuNS@g-CN/anti-Tau/BSA electrode was incubated with Tau protein, the ECL intensity decreased significantly due to the steric hindrance of Tau protein (yellow curve). Without grafting of the anti-Tau step on the electrode, whenever it was incubated with only Tau protein, the cathodic ECL dropped slightly (purple curve), confirming the effective interactions between Tau protein and anti-Tau. These results confirm the successful construction of the ECL sensor.

To investigate the effect of sensor construction steps on the electrode, the cyclic voltammetry (CV) voltammograms were recorded for each stage in the presence of redox standard (details are provided in the ESM). As illustrated in Figure S3, a clear redox peak (black curve) with the use of appeared when the bare GC electrode. The redox peak current decreased moderately after the immobilization of the AuNSs@g-CN nanostructure (red curve) on the electrode suggesting the high conductivity and active surface area of the AuNSs@g-CN nanostructure. When anti-Tau (blue curve) and Tau protein (green curve) were consecutively immobilized on GCE/AuNSs@g-CN, the redox peak currents diminished considerably due to the resistance of anti-Tau antibody and Tau protein [32].

### 3.3. Optimization of Experimental Parameters

Some experimental parameters were optimized to obtain high sensitivity in the ECL determination of Tau. The five main optimized parameters include film formation temperature of the AuNSs@g-CN nanostructure on cleaned GCE, buffer concentration, Tau antibody concentration, immune reaction time and temperature between the antigen (Tau protein), and the antibody (Tau antibody).

In our previous work, the optimum pH value for the GCE/AuNS@g-CN system, amount of AuNSs@g-CN nanosheets, and the K_2_S_2_O_8_ solution concentration were obtained at 7.5, 10 µL, and 0.1 M, respectively.

To obtain a high and stable ECL emitter, the temperature for film formation of the AuNSs@g-CN nanostructure on cleaned GCE was studied within the range of 25–45 °C. As illustrated in Figure S4, the ECL intensity increased with increasing the temperature and finally reached a plateau at 40 °C; therefore, it was selected as the optimum film-forming temperature.

The ECL intensity enhanced significantly with increasing the concentration of PBS from 0.03 M to 0.15 M (Figure S5) and then remained constant beyond 0.1 M. Therefore, the 0.10 M PBS solution was used for all experiments.

A sufficient amount of antibody ensures the complete target analyte capturing on the biosensor interface. As displayed in Figure S6, the ECL intensity decreased significantly with increasing antibody concentrations and then reached a plateau at 7. 14 ng mL^−1^, indicating immobilized antibody saturation.

Incubation temperature has a significant influence on antibody and antigen reaction kinetics. According to Figure S7, for a specific amount of antigen, at the incubation temperature of 37 °C, the ECL response was maximum which was chosen for the next tests.

The investigation of incubation time for the reaction of antibody and antigen (Tau protein) reaction revealed that and 50 min was enough for the completion of the immune reaction (Figure S8).

**Figure 3:**
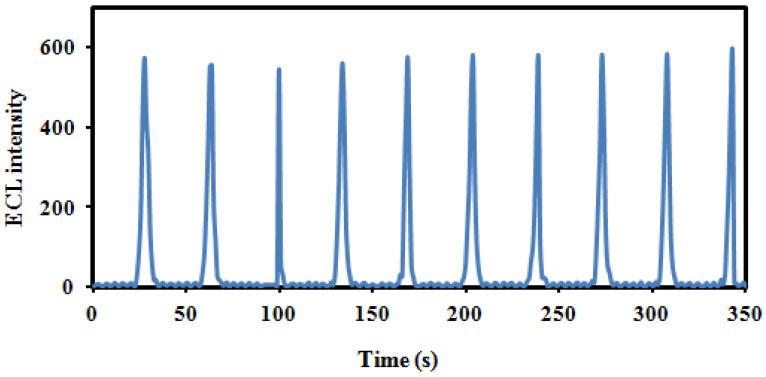
The stability of ECL response under 10 cycles of consecutive cyclic potential scan obtained from GCE/AuNS@g-CN/anti-Tau/BSA/Tau protein system.

The stability of the ECL emitting materials is the most important parameter for analytical application. Figure 3 show the ECL stability of the immunosensor response for detecting 1 ng/mL of Tau protein. Under 10 cycles of a successive cyclic potential scan, the ECL intensity was extremely stable, and the RSD was 3.3. Furthermore, when the GCE/AuNS@g-CN immunosensor was stored at 4 °C, it was stable for more than 2 weeks, and the ECL intensity was 97.4% of the original ECL value after 2 weeks.

### 3.4. Analytical Performance

**Figure 4:**
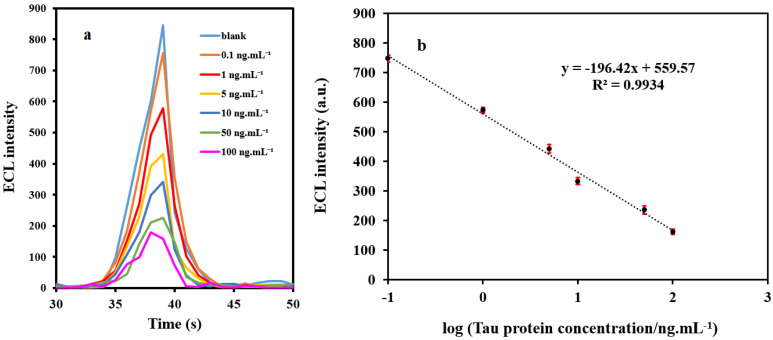
(**a**) The ECL response of immunosensor for various concentrations of Tau protein, and (**b**) Calibration curve. Error bars show the standard deviations for three replicates. (concentration of K_2_S_2_O_8_: 0.1 M, pH = 7.5, and concentration of PBS: 0.1 M).

Under the optimized conditions, the ECL intensity of the immunosensor decreased linearly with the increase of the Tau protein concentration (Figure 4a). The calibration plot (Figure 4b) displays an excellent linear response over the range of 0.1–100 ng mL^−1^. The regression equation was Y = −196.42X + 559.57 whit a correlation coefficient of 0.9934, where Y was ECL intensity and X was the logarithm of the concentration of Tau protein (ng mL^−1^). The LOD for Tau protein at a signal-to-noise ratio of 3 (S/N = 3) was 0.034 ng mL^−1^ which is less than levels of tau protein in serum samples [33]. These results highlight that this ECL method can be successfully utilized to detect the crucial biomarker of AD in plasma at low picomolar levels. Details of the percentage of variation in ECL intensity are provided in Figure S9.

**Figure 5:**
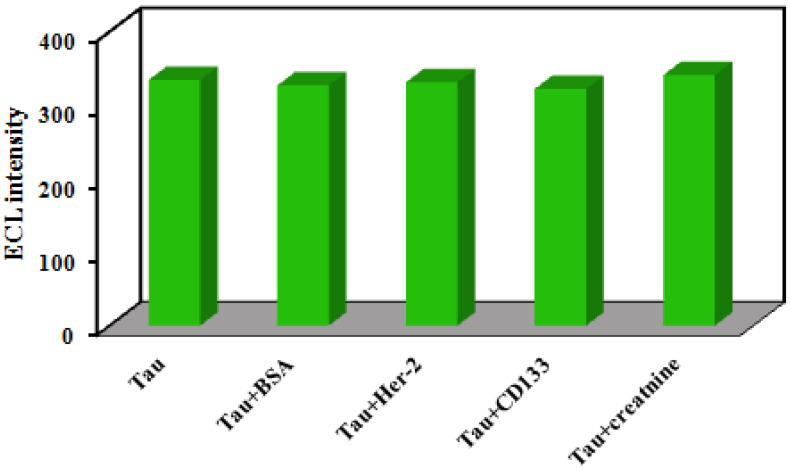
The ECL response toward different possible interferences (concentration of interferences 100 ng mL^−1^ in the presence of 1 ng mL^−1^ Tau protein).

The specificity of the developed immunosensor for Tau protein determination was investigated to survey its feasibility. Some possible interferences containing BSA, creatinine, HER-2, and CD133 peptide were added to 1 ng mL^−1^ of the Tau protein solution with a concentration 100 times higher than Tau protein (the concentration of interferences were 100 ng mL^−1^). As displayed in Figure 5, the ECL response of the immunosensor for Tau in the presence of interferences varied less than 5% of the response to pure Tau, suggesting that the immunosensor has admissible specificity for the quantification of Tau protein in serum samples.

The performance of the ECL immunosensor for the detection of Tau protein in real serum samples was assayed by testing the specificity of the ECL immunosensor to potential interfering materials. A solution containing 1 ng mL^−1^ of tau protein and excess amounts of interfering materials was prepared for each interfering material, such as common ions (Na^+^, K^+^, Ca^2+^, Mg^2+^, Zn^2+^, Fe^3+^) and some biomolecule (alanine, glycine, tryptophan, glucose, lactose and sucrose). Subsequently, its ECL signal was obtained. The tolerance limit was taken as the concentration of the added species causing less than 5% relative error. The tolerable molar ratios were over 100-fold for all tested interferents.

We further examined the accuracy of the ECL immunosensor using the area under the receiver operating characteristic (ROC) curve (AUC), which is a general indication of accuracy in a diagnostic test. The AUC of our ECL immunosensor was 0.972 (Figure S10), indicating the favorable performance of this method.

The feasibility of the ECL immunosensor for the quantification of Tau protein biomarker in serum samples was examined. Healthy human serum samples were spiked with different concentrations of Tau protein, and then recovery studies were carried out. As shown in Table 1, the ECL immunosensor displayed a good outcome (recoveries are in the range of 94.48% to 108%, and all RSDs are lower than 5%), indicating that the proposed ECL immunosensor provides a facile and inexpensive procedure for monitoring and predicting of AD-using serum samples.

## 4. Conclusions

For the first time, an ECL immunosensor is reported for the screening of Tau protein (AD marker) in serum samples. CN nanosheets were decorated with AuNSs and used as excellent ECL luminophores. In this system, small-sized AuNSs on the surface of g-CN nanosheets played an important role in amplifying the ECL intensity and immobilizing the antibody as an immunosensor recognition element. The resulting immunosensor illustrated excellent sensitivity and great analytical performance and was successfully utilized for the quantification of Tau protein in real human serum samples. In the future, similar non-spherical gold nanoparticle enhanced ECL sensors can be designed for detecting other trace biomolecules.

## Figures and Tables

**Table 1:** Analytical results of the determination of Tau protein in human serum samples using the proposed biosensor.

Sample	Added (ng/mL)	Found	Recovery (%)
	50	47.24	94.48
Serum	10	9.57	95.7
	5	5. 33	106.6
	1	1. 08	108

## Data Availability

Not applicable.

## Supplementary Materials

The following are available online, Materials, Synthesis of gold nanostars and gold nanoparticls; Figure S1. TEM image of AuNP@g-CN nanostructure; Figure S2. UV-Vis absorption spectra of colloidal solution of AuNSs and AuNSs@g-CN nanosheets; Figure S3. CV curves for bare GCE electrode at difreent steps of sensor constructio; Figure S4. The effect of the temperature on draying of AuNSs@g-CN nanostructure; Figure S5. The effect of PBS concentration on the ECL intensity of GCE/AuNSs@g-CN; Figure S6. Effect of anti-Tau concentration on the ECL intensity; Figure S7. Effect of temperature on the anti-Tau -Tau protein reaction kinetics; Figure S8. Effect of incubation time on the anti-Tau -Tau protein reaction; Figure S9. ECL sensor signal change percentage; Figure S10. Receiver operating characteristic (ROC) curve.

## Sample Availability

Samples of the compounds AuNP@g-CN nanostructure is available from the authors.
