# Supplementary material for: An Electrochemiluminescence Biosensor for the Detection of Alzheimer’s Tau Protein Based on Gold Nanostar Decorated Carbon Nitride Nanosheets
Source: Molecules. 2022 Jan 10;27(2):431. doi: 10.3390/molecules27020431 (PMC8779933; doi:10.3390/molecules27020431)
Supplement: Supplementary file 1 [file molecules-27-00431-s001.zip › molecules-1534424-supplementary.pdf]

molecules

MDPI

Article

An Electrochemiluminescence Biosensor for the Detection of Alzheimer's Tau Protein Based on Gold Nanostar Decorated Carbon Nitride Nanosheets

Roghayeh Jalili [1], Salimeh Chenaghlou [1], Alireza Khataee [1,2,3,*], Balal Khalilzadeh [4] and Mohammad-Reza Rashidi [4,*]

1 Research Laboratory of Advanced Water and Wastewater Treatment Processes, Department of Applied Chemistry, Faculty of Chemistry, University of Tabriz, Tabriz 51666-16471, Iran; r_jalili2010@yahoo.com or r.jalili@tabrizu.ac.ir (R.J.); salimehchenaghlou@gmail.com (S.C.)
2 Department of Environmental Engineering, Gebze Technical University, Gebze 41400, Turkey
3 Department of Material Science and Physical Chemistry of Materials, South Ural State University, 454080 Chelyabinsk, Russia
4 Stem Cell Research Center (SCRC), Tabriz University of Medical Sciences, Tabriz 51666-14711, Iran; khalilzadehb@tbzmed.ac.ir
* Correspondence: a_khataee@tabrizu.ac.ir (A.K.); rashidi@tbzmed.ac.ir (M.-R.R.)

Materials

All reagents were of analytical reagent grade and used without further purification. Melamine, polyvinylpyrrolidone (PVP), Potassium persulfate ($K_2S_2O_8$), sodium borohydride, ($NaBH_4$, 99%) and bovine serum albumin (BSA) were obtained from Sigma-Aldrich (USA) and used as received. Chloroauric acid ($HAuCl_4$) was purchased from Alfa Aesar (Karlsruhe, Germany), N, N-dimethylformamide (DMF), Creatinine (Sigma-Aldrich), HER-2 protein (Abcam), CD133 protein (Biorbyt, USA) and KCl were used to prepare $[Fe(CN)_6]^{3−/4−}$ solution. Mouse Anti-Phosphorylted-Tau (pThr231) was obtained from Ray biotech (Mexico). Tau peptide ($Thr(PO_3H_2)$) was purchased from Bachem (Bubendorf, Switzerland). Healthy human serum samples were kindly provided by Drug applied research center, Tabriz University of Medical Sciences (Tabriz, Iran). The samples were spiked with different levels of Tau protein (50, 10, 5, and 1 ng mL^{-1}) and analyzed without further pretreatment.

Synthesis of Gold Nanostars and Gold Nanoparticls

Gold nanostars (Au NSs) were synthesized via a two-step seed-mediated growth method. Firstly, 2–3 nm Au seeds were synthesized. In detail, 22 μL of 0.1136 M $HAuCl_4$ aqueous solution was added to a 47.5 mL solution of 0.098 g polyvinylpyrrolidone (PVP, MW10000) in DMF/H_2O (18:1 v/v). Then 2.5 mL of 10 mM prepared ice-cold $NaBH_4$ solution was added immediately and keep stirring for another 2 h. The resultant solution (seed solution) was kept at room temperature overnight before use. In the second step (growth), 82 μL of 50 mM $HAuCl_4$ was added to 15 mL of 10 mM PVP solution in DMF. Then 20 μL of seed solution was injected into the mixture rapidly and stirred for 2 h. It changed from colorless to blue, suggesting the formation of AuNSs. The resultant blue solution was centrifuged, the supernatant was decanted, and the AuNSs were re-dispersed in ultrapure water. For synthesis of AuNPs, a 100 mL 1% $HAuCl_4$ aqueous solution was injected into a round-bottom flask fitted with a reflux condenser under vigorous boiling and stirring. 5 mL of 1% trisodium citrate solution was added rapidly to the above boiling solution. Under reflux, the solution was stirred for another 20 min and the color of the solution changed from pale yellow to deep red during the time. The solution was cooled to room temperature and then stored at 4 °C in the refrigerator.

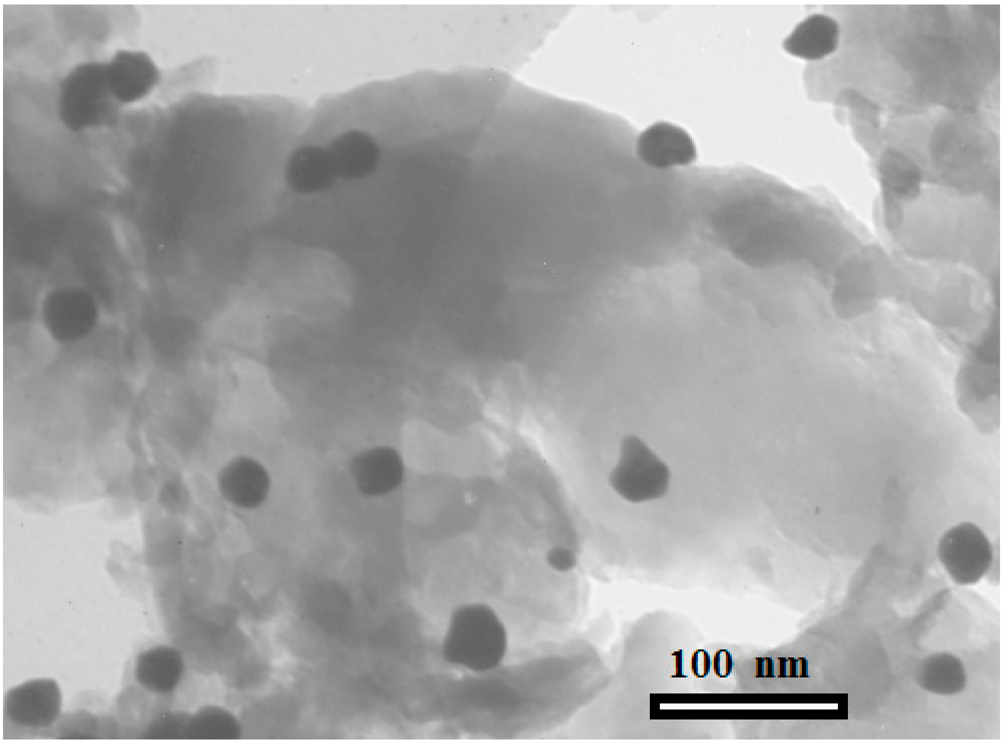

Figure S1. TEM image of AuNP@g-CN nanostructure.

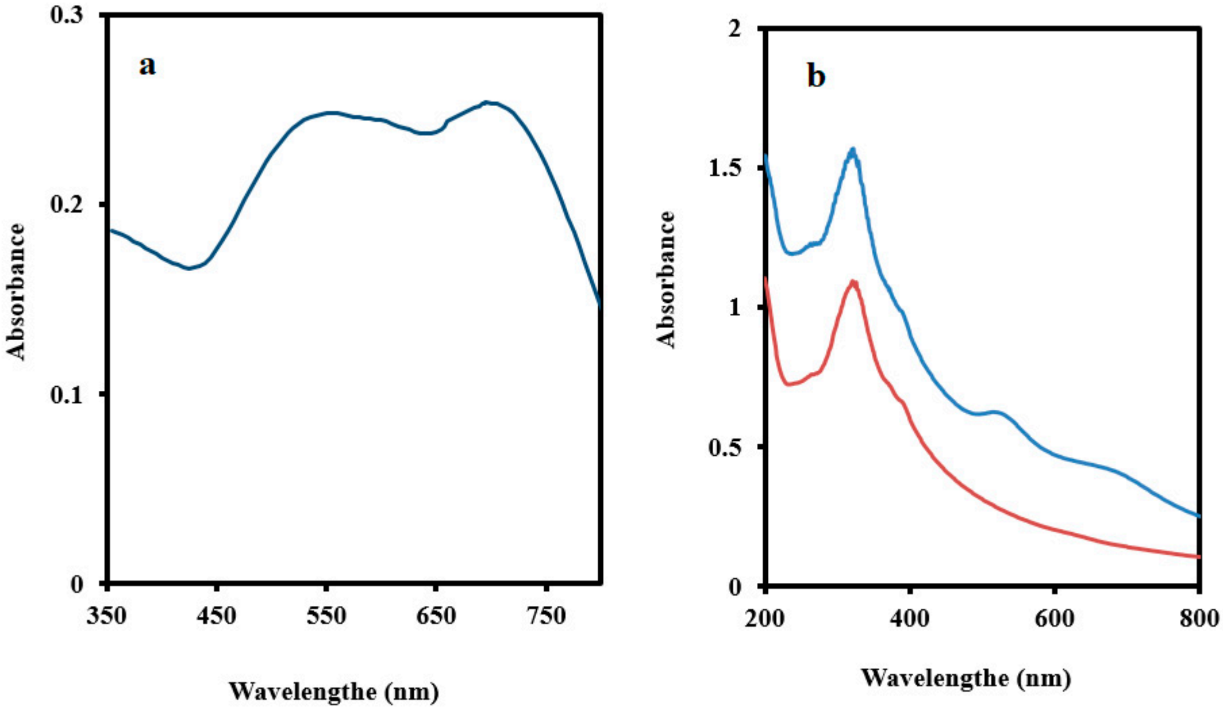

Figure S2. **(a)** UV-Vis absorption spectra of colloidal solution of AuNSs. **(b)** g-CN nanosheets (red curve) and AuNSs@g-CN nanosheets (blue curve).

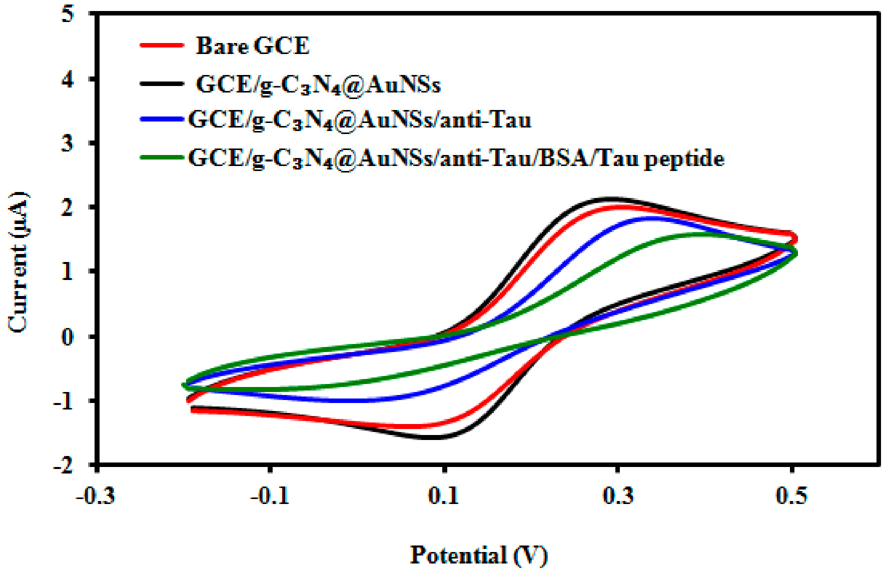

Figure S3. CV curves for bare GCE electrode (black curve), GCE/AuNSs@g-CN nanosheet system (red curve), GCE/AuNSs@g-CN/ anti-Tau system (blue curve) and GCE/AuNSs@g-CN/anti-Tau/BSA/Tau protein system (green curve) in 0.5 M KCl containing 5 mM ($[Fe(CN)_6]^{4-}/[Fe(CN)_6]^{3-}$) as redox standard.

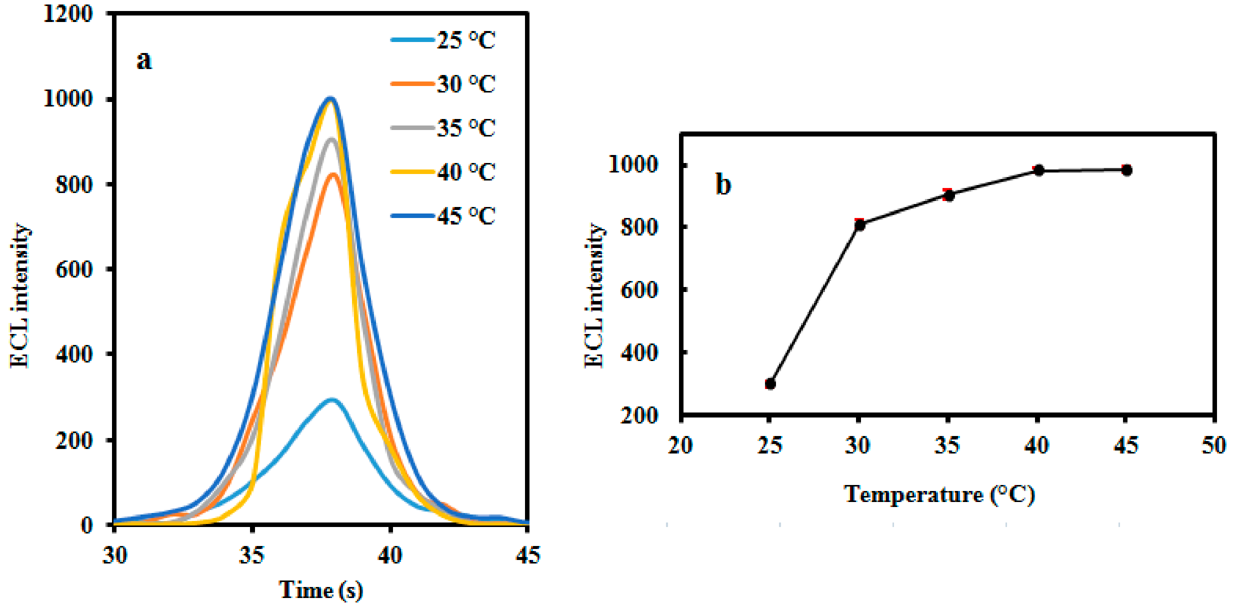

Figure S4. (a) The effect of the temperature on draying of AuNSs@g-CN nanostructure after dropcasting on the cleaned GCE and (b) variation of the ECL intensities versus temperature of film formation. (Concentration of $K_2S_2O_8$: 0.12 M, pH= 7.5, and concentration of PBS: 0.1 M)

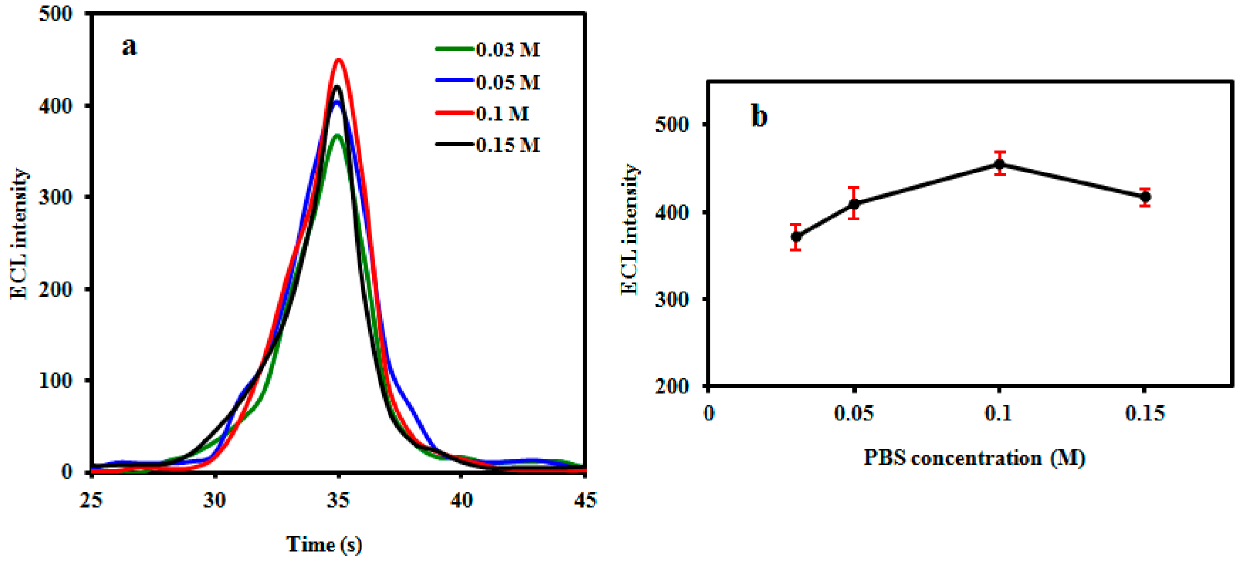

Figure S5. (a) The effect of PBS concentration on the ECL intensity of GCE/AuNSs@g-CN system and (b) variation of the ECL intensities versus different concentrations of PBS. (Concentration of $K_2S_2O_8$: 0.1 M, and pH= 7.5.)

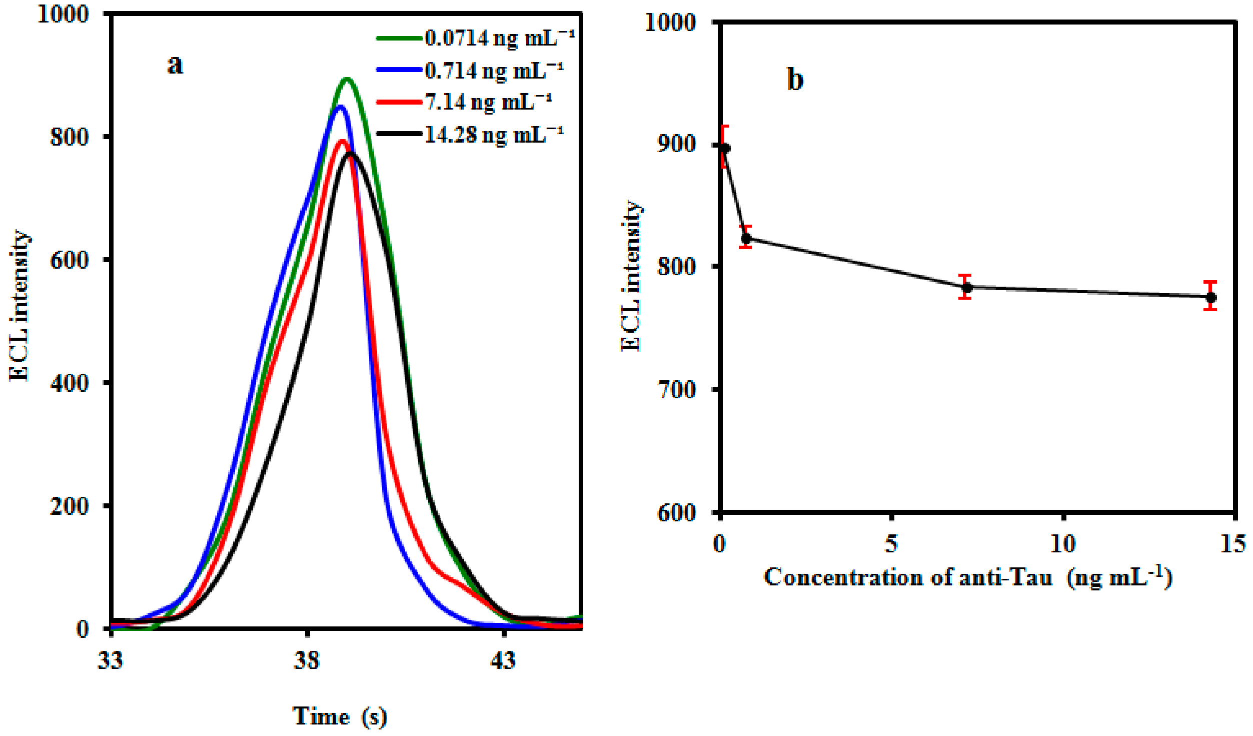

Figure S6. (a) Effect of anti-Tau concentration on the ECL intensity of GCE/AuNSs@g-CN/anti-Tau system and (b) variation of the ECL intensities versus different concentrations of anti-Tau. (Concentration of $K_2S_2O_8$: 0.1M, pH= 7.5, concentration of PBS: 0.1 M, incubation temperature: 4 °C, and incubation time: 12 h)

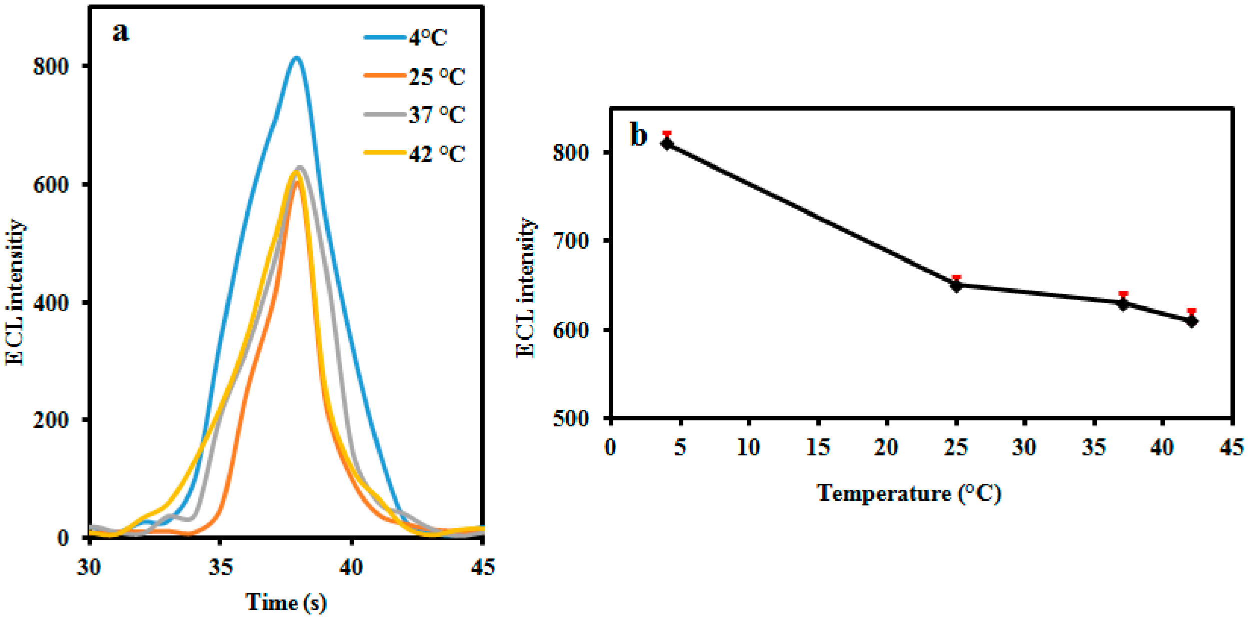

Figure S7. (a) Effect of temperature on the anti-Tau -Tau protein reaction kinetics and (b) variation of the ECL intensities versus different temperature of anti-Tau -Tau protein. (Concentration of $K_2S_2O_8$: 0.1M, pH= 7.5, concentration of PBS: 0.1 M, concentration of anti-Tau: 7.14 ng mL^{-1}, concentration of Tau protein: 1 ng mL^{-1}, and incubation time: 1h)

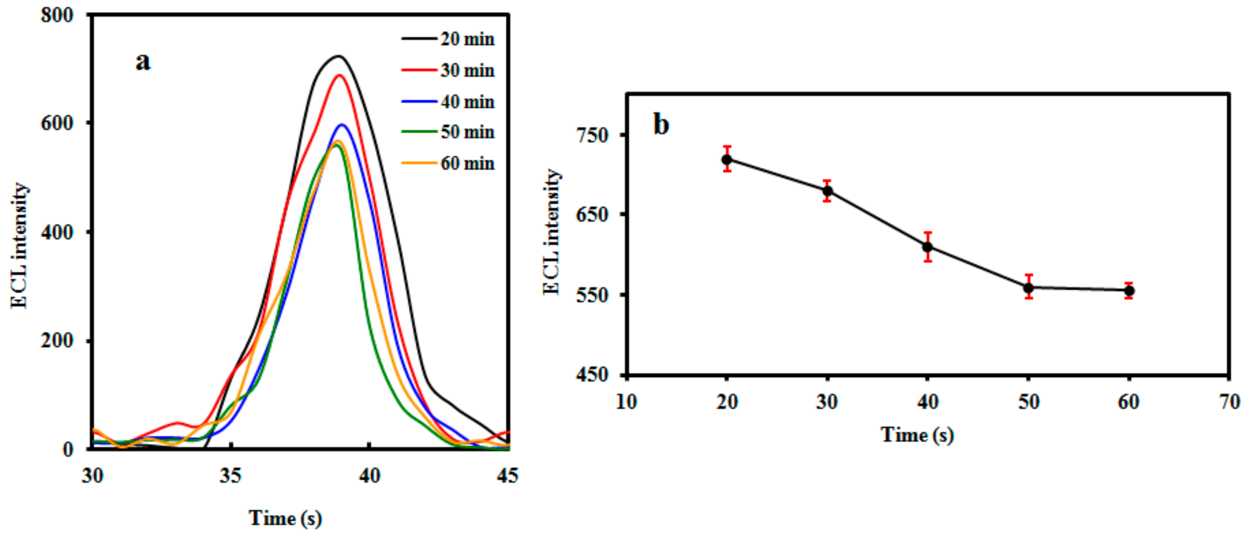

Figure S8. (a) Effect of incubation time on the anti-Tau -Tau protein reaction and (b) variation of the ECL intensities versus different incubation times of anti-Tau -Tau protein reaction. (Concentration of $K_2S_2O_8$: 0.1M, pH= 7.5, concentration of PBS: 0.1 M, concentration of anti-Tau: 7.14 ng mL^{-1}, and concentration of Tau protein: 1 ng mL^{-1}).

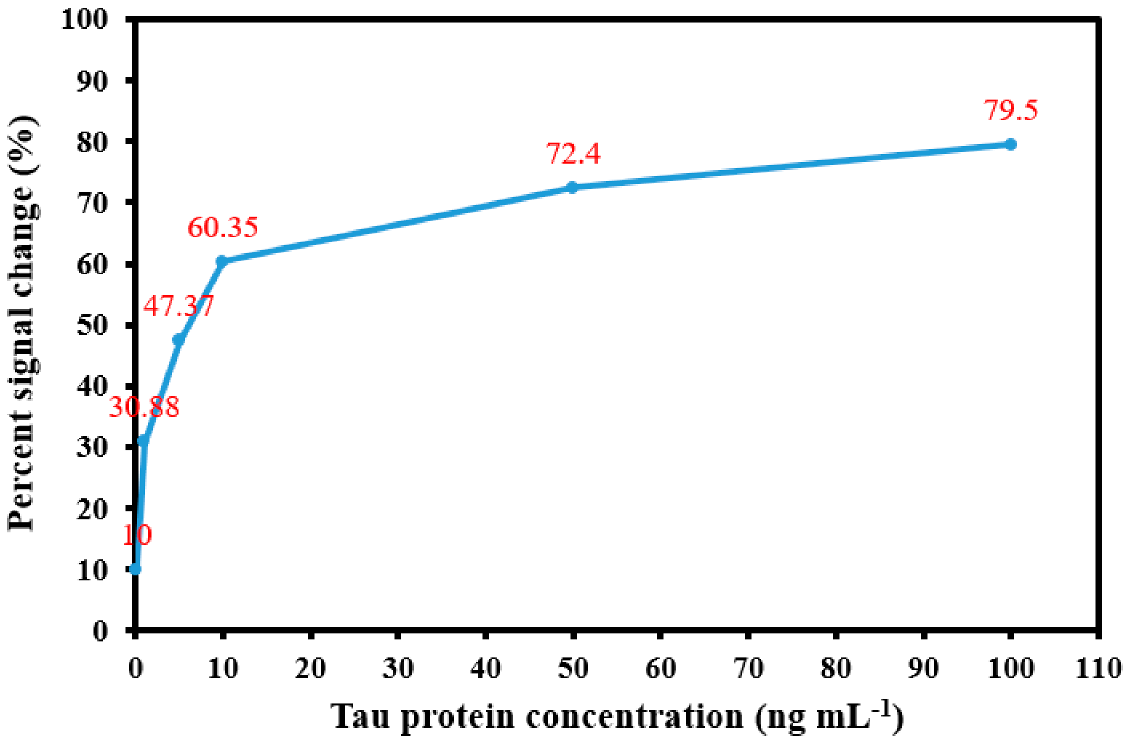

Figure S9. ECL sensor signal change percentage. To quantify ECL sensor response, we use the traditionally used "percent signal change" defined as: (I0-I / I0) × 100, where I0 and I represent the ECL intensity in the absence and presence of analyte, respectively.

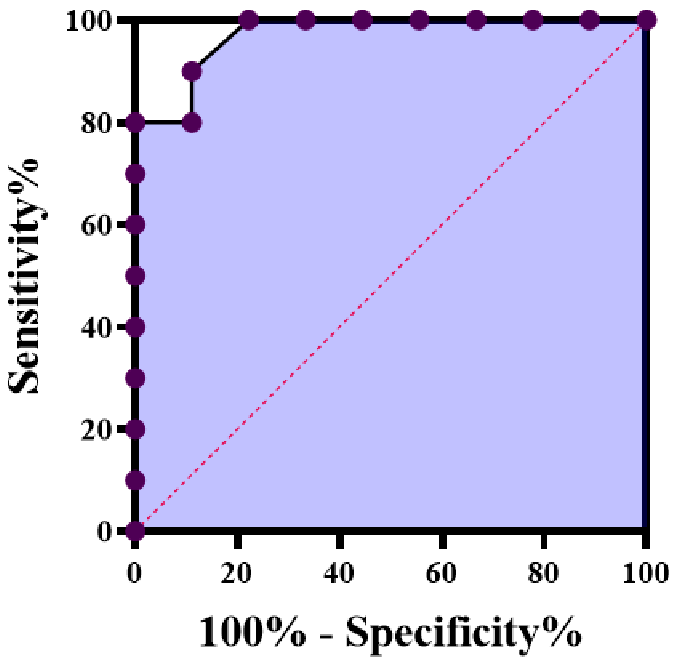

Figure S10. Receiver operating characteristic (ROC) curve.
